# Disparities in Diagnosis, Management, and Outcomes of Hepatocellular Carcinoma: A Review of Global and Sociodemographic Factors

**DOI:** 10.1007/s12029-026-01428-8

**Published:** 2026-03-17

**Authors:** Cathy Zheng, Manuela Araque, Anthony Quiej, Keri-Ann Buchanan-Peart, Patricia D. Jones

**Affiliations:** 1 University of Miami, Jackson Memorial Hospital Internal Medicine Residency, Miami, FL USA; 2 Department of Medicine, Division of Digestive Health and Liver Diseases, University of Miami Miller School of Medicine, Miami, FL USA; 3 Department of Public Health Sciences, Sylvester Comprehensive Cancer Center, Miami, FL USA; 4 Sylvester Comprehensive Cancer Center, University of Miami Miller School of Medicine, Miami, FL USA

**Keywords:** Hepatocellular carcinoma, Disparities, Health equity

## Abstract

Hepatocellular carcinoma (HCC), the most common primary liver cancer, is a leading cause of cancer-related death. There are significant disparities in risk, receipt of screening for HCC, stage at diagnosis, receipt of treatment and survival. Differential HCC risk for HCC is driven by variation in the etiology of underlying liver disease, which varies by race, ethnicity, gender, and geographic location. Sociocultural and structural forces influence access to health care, which may limit adherence to guideline-recommended HCC screening intervals. Black race and uninsured status are associated with decreased likelihood of receiving HCC screening. These disparities in screening impact stage at presentation as patients who are diagnosed through screening present at earlier stages of disease compared to patients who are diagnosed due to symptoms. Socioeconomic status, geography, race, and ethnicity are all associated with receipt of treatment and survival after HCC diagnosis. Guided by the National Institute on Minority Health and Health Disparities (NIMHD) Research Framework, this work aims to present a comprehensive overview of disparities in HCC risk, screening, diagnosis, treatment, and outcomes.

## Introduction

**Fig. 1:**
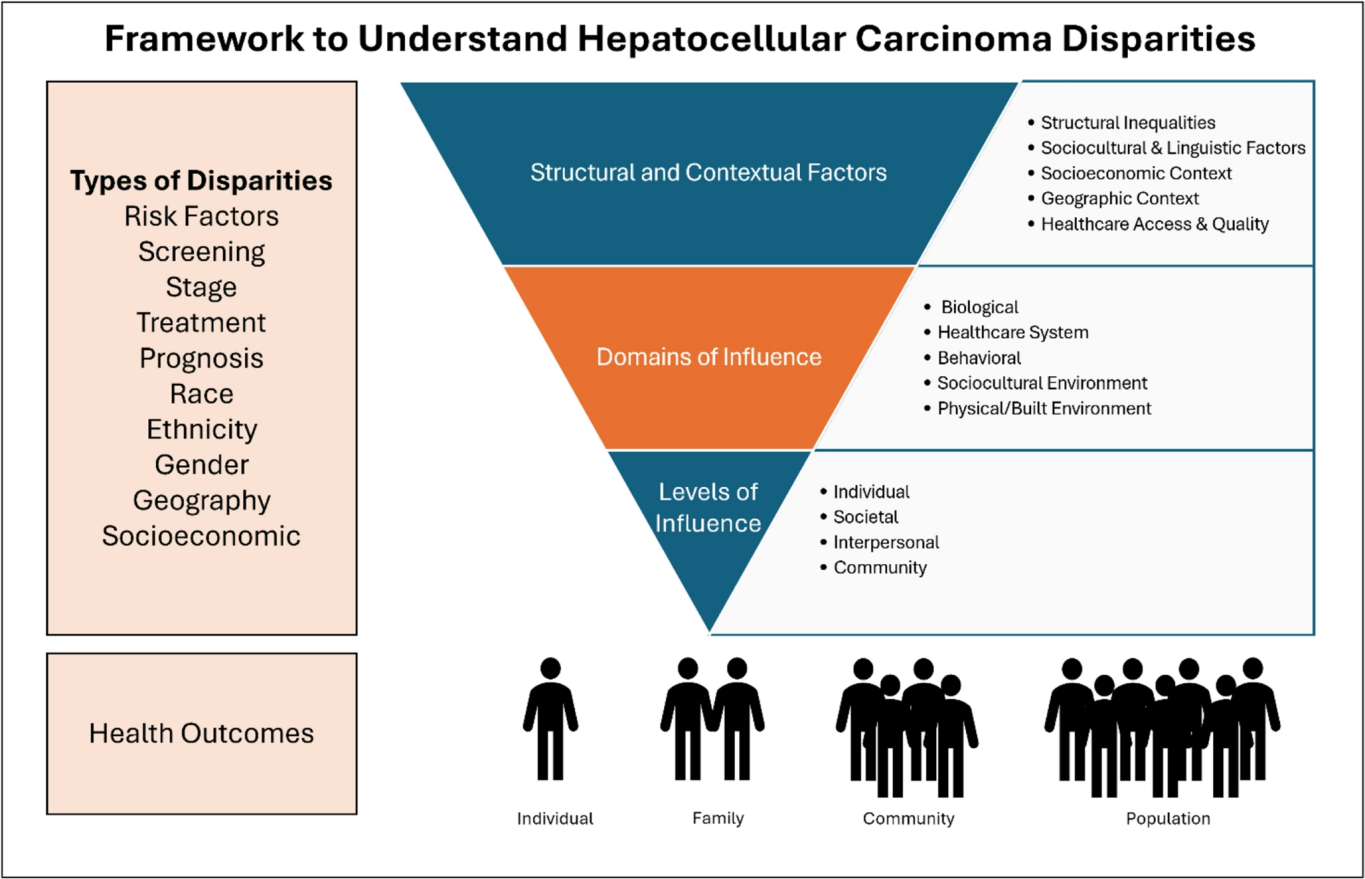
Disparities in Hepatocellular Carcinoma (HCC) are driven by structural and contextual factors across levels and domains and influence health outcomes at the individual, family, community, and population level. Adapted from the National Institute on Minority Health and Health Disparities (NIMHD) Research Framework.

The risk of developing hepatocellular carcinoma is driven by distinct global patterns of chronic liver disease. In the United States, geographic and sociodemographic factors including sex, race, and ethnicity, contribute to differential HCC risk. There are notable disparities in HCC screening, diagnosis, treatment, and outcomes which occur across various levels and domains of influence. The National Institute on Minority Health and Health Disparities (NIMHD) Research Framework [1] is a useful tool to address the multifaceted nature of disparities. This review focuses on sociocultural and health care system domains and outcomes at the individual and population levels. **See** Fig. 1. We organize this review of disparities in risk, screening, stage at diagnosis, treatment, and outcomes by highlighting global and geographic factors, racial and ethnic disparities, gender considerations, and socioeconomic factors.

## Disparities in HCC Risk

### Global Burden and the Shifting Etiology of HCC

Hepatocellular carcinoma (HCC) is the sixth most frequently diagnosed cancer worldwide and the third leading cause of cancer-related mortality [2, 3]. It is challenging to quantify the overall burden of HCC; Global Cancer Statistics 2020 (GLOBOCAN 2020) reported 905,700 incident cases and 830,200 deaths from HCC in 2020. The Global Burden of Disease Study 2021 (GBD 2021) reported 519,986 incident cases and 475,269 deaths from liver cancer [3–5]. Worldwide, HCC incidence is highest in regions of Asia and Africa[6], specifically Micronesia (age-standardized incidence rate (ASIR) 15.1/100,000), Eastern Asia (ASIR 14.7/100,000), and Northern Africa (ASIR 14.2/100,000)[7]. Over 70% of HCC cases occur in Asia [8] with China alone accounting for over 50% of the global burden [6]. Africa accounts for 7.8% of liver cancer cases worldwide, of which 77% are from HCC [8]. 

**Fig. 2:**
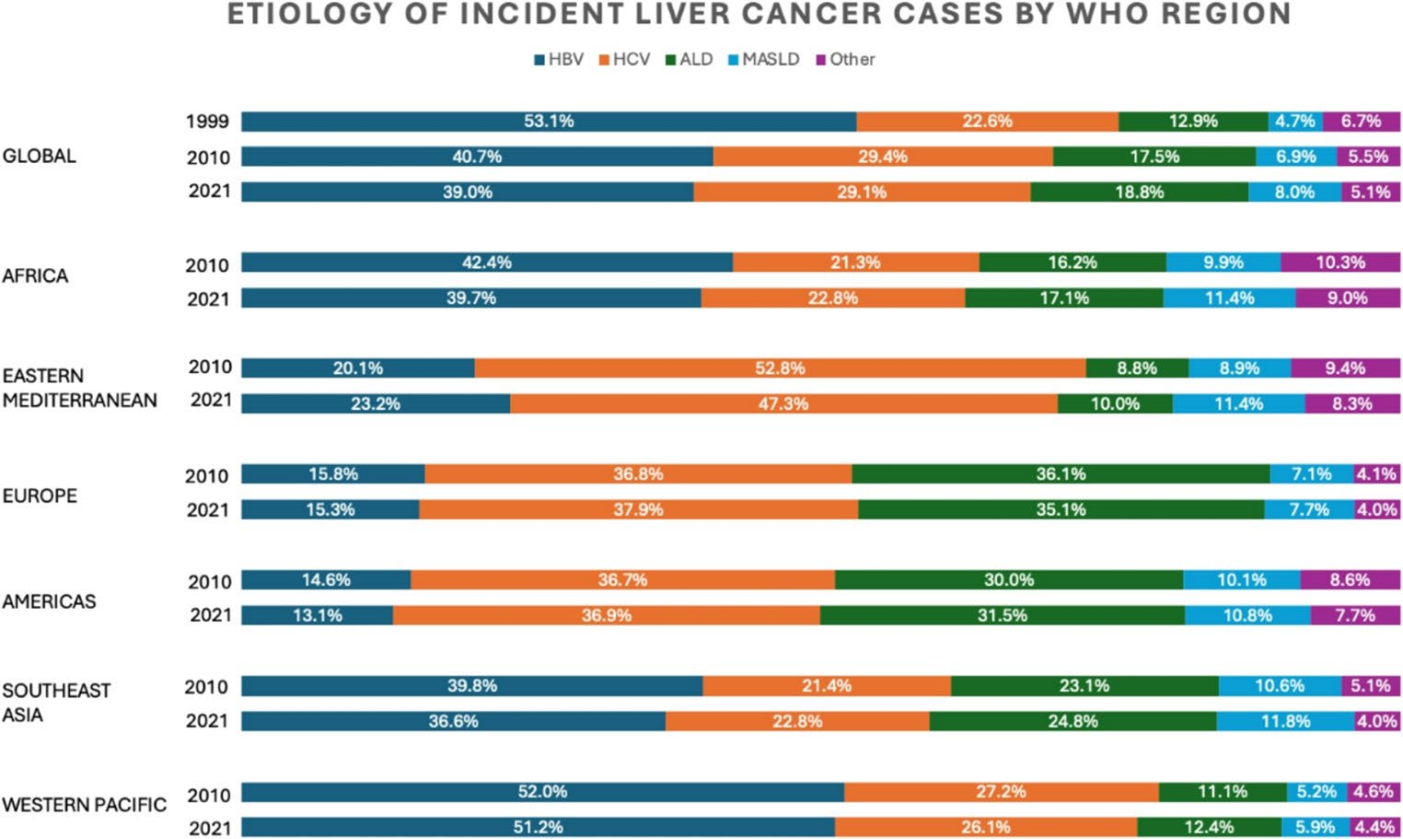
Shifting epidemiology of underlying etiology of incident liver cancer cases by World Health Organization (WHO) Region. Figure created using data from Tan EY, Danpanichkul P, Yong JN, et al. Liver cancer in 2021: Global Burden of Disease study. *J Hepatol*. 2025;82(5):851–860. Doi:10.1016/j.jhep.2024.10.031 and Singal AG, Kanwal F, Llovet JM. Global trends in hepatocellular carcinoma epidemiology: implications for screening, prevention and therapy. *Nat Rev Clin Oncol*. 2023;20(12):864–884. Doi:10.1038/s41571-023-00825-3

HCC occurs nearly universally in the context of chronic liver disease or cirrhosis. Globally, the population attributable fraction (PAF), which measures the extent to which individual risk factors contribute to HCC burden, is 56% for hepatitis B virus (HBV), 26% for alcohol, 20% for hepatitis C virus (HCV), 17% for aflatoxin, 9% for obesity, and 7% for diabetes [3, 9–13] The prevalence of various risk factors has shifted over time due to HBV vaccination programs, increased HCV treatment and cure rates, and increased prevalence of metabolic risk factors, though with marked regional variation. Among HCC cases diagnosed worldwide in 1990, 53.1% were HBV-related, 22.6% HCV-related, 12.9% alcohol-related, 4.7% related to metabolic dysfunction-associated steatotic liver disease (MASLD), and 6.7% from other causes [8]. By 2019, the corresponding estimates were 41.0%, 28.5%, 18.4%, 6.8%, and 5.3%[8]. See Fig. 2. Beyond global variations in HCC etiology, geographic, racial/ethnic, gender, and socioeconomic disparities contribute to overall disparities in HCC risk.

### Geographic Disparities in HCC Burden

Data from the US Cancer Statistics Registry, which covers over 90% of the population, showed the highest HCC age-adjusted incidence rates (AAIR) in the Southern and Western US. In 2012, Texas and Hawaii had the highest AAIR (9.71 and 9.68, respectively) [14]. There are notable disparities in HCC incidence when comparing urban and rural settings. A Surveillance, Epidemiology and End Results (SEER) analysis from 2004 to 2017 revealed higher HCC incidence rates in large metropolitan areas (population exceeding 1 million), with rates ~ 1.5 times higher than non-metropolitan or rural areas [15]. Similar rural-urban disparities are observed in other countries. A study in Henan, China found that the risk of developing liver cancer is about 1.1 times greater in urban compared to rural areas [16]. Higher HCC incidence is also associated with major city residence in Australia[17].

In a southeastern US cohort, urban residency was associated with increased HCC risk among Black (HR = 1.64, 95% CI 1.09–2.45) and White individuals (HR = 2.34, 95% CI 1.17–4.65) compared to rural residency [18]. In contrast, American Indian/Alaska Native (AI/AN) individuals living in nonmetropolitan or rural regions had the highest HCC incidence (rate ratio = 1.20, 95% CI 1.03–1.40) compared to metropolitan regions [15]. However, incidence is rising in these non-metropolitan and rural areas. Between 2004 and 2017, the greatest average annual percent change (APC) in HCC incidence occurred in nonmetropolitan/rural areas for both men (APC 4.42) and women (APC 4.90), significantly exceeding increases observed in metropolitan areas (APC 0.97 in men and APC 1.45 in women) [15]. 

### Racial and Ethnic Disparities in HCC Risk

HCC risk is significantly higher in American Indian/Alaska Native, Hispanic, Asian and Black populations, compared to White populations [19–24]. The most recent SEER-22 data from 2019 reports ASIR of 15.5 in Hispanic populations, 14.6 in AI/AN, 12.1 in Asian/Pacific Islander (API), 10.7 in Black, and 6.9 in White populations [25]. There are interesting epidemiologic trends in HCC risk within racial groups. US-born Black individuals had higher incidence rates (AAIR: men 13.1; women 3.9), compared to those born in Haiti (AAIR: men: 8.0; women: 2.2) or the West Indies (AAIR: men: 3.0; women: 1.6)[26]. Among Hispanic men, the AAIR was highest among Puerto Rican men (19.4), followed by US-born Mexican (17.1), Central American (10.8), Dominican (9.4), foreign-born Mexican (9.2), South American (8.1), with the lowest rate in Cuban (7.4) men [27]. However, the AAIR in Hispanic women were similar, ranging from 4.4 in Central American women to 5.4 in foreign-born Mexican women. The lowest incidence rate was in Cuban women at 2.1[27]. Similarly, Southeast Asian individuals (which included Vietnamese, Laotian, and Cambodian individuals) had nearly twice the HCC incidence rate of Korean individuals, the API subgroup with the next highest HCC incidence rate[28]. In California, Southeast Asian individuals had an 8 to 9-fold higher HCC risk compared to non-Hispanic White individuals [29]. 

### Etiologies of HCC Vary by Race/Ethnicity

Recent studies have examined differences in HCC etiologies by race and ethnicity; HCV-related HCC is more common among US-born Puerto Rican, African American, and Mexican men compared to non-Hispanic White men[27]. This finding reflects many contextual factors, including higher incarceration rates among US-born non-White individuals [30]. Metabolic risk factors are also unevenly distributed with higher prevalence of metabolic conditions and risk of HCC progression in Hispanic, Black, and American Indian populations [21, 31]. Compared to non-Hispanic White men and women, MASLD-related HCC rates were about 50% higher in Hispanic men and twice as high in Hispanic women[30]. Rates of ALD-related HCC were highest among American Indian and US-born Latino individuals [30]. Genetic factors implicated in HCC may contribute further to these disparities; including variants of *PNPLA3* and *PPP1R3B* genes in Hispanic individuals, and *PNPLA3*,* NCAN*,* GCKR*, and *PPP1R3B* genes in Black individuals [19]. Among Asian individuals, HBV is the leading cause of HCC in Chinese, Korean, and Southeast Asian American individuals, whereas HCV predominates among Japanese Americans and MASLD-related HCC is most prevalent among Filipino (men: 6.2; women: 2.5) and Pacific Islander (men: 6.0; women: 2.9) individuals [30]. 

### Gender Disparities

Globally, men have 2- to 4-fold higher incidence rates compared to women, with the largest disparities observed in Europe [4, 9]. In other countries, the disparity is less pronounced [9]. These global gender differences in HCC risk are likely related to differential exposure to risk factors and intrinsic biologic differences. The odds ratio for HBV-related HCC risk is over twice as high in men (odds ratio (OR) 28.2, 95%CI = 22.3–35.6) compared to women (OR 13.2, 95%CI = 9.5–18.3)[32]. Men with MASH have a 2- to 7-fold higher risk of developing HCC compared to women; the disproportionate effect of metabolic liver disease in men may be related to differences in fat distribution, insulin resistance, and inflammatory responses [33, 34]. Risk factors such as alcohol consumption, tobacco use, and occupational exposure to hepatotoxic chemicals are more prevalent in men, but persistent sex differences despite shifting exposures suggest roles for intrinsic biological factors e.g. hormonal mechanisms[21, 33–35].

### Socioeconomic Factors Contributing to HCC Risk

At the individual level, low-socioeconomic status (SES) is associated with 1.2 to 1.5 times higher HCC incidence rates with the greatest disparities among Black individuals [36]. In the US, individuals with a median household income below $40,000 had 1.17 times greater HCC incidence compared with income above$70,000 [15]. Contextual or neighborhood-level factors also affect risk; low neighborhood income, limited access to nutritious food options and green spaces, economic instability, lack of employment, and low health literacy due to reduced educational opportunities all contribute to disparities in HCC risk and may limit access to health care, which hinders screening for HCC[37–40].

##  Disparities in HCC Screening

Patients with risk factors for HCC, including cirrhosis and chronic HBV, should undergo semiannual screening with ultrasound ± alpha-fetoprotein, per the American Association for the Study of Liver Diseases (AASLD) [41] guidance and the European Association for the Study of the Liver [42] clinical practical guidelines. However, HCC screening recommendations vary slightly by global region [43, 44]. Screening in non-cirrhotic patients with chronic HCV and advanced (F3) liver fibrosis is recommended by EASL and the Japan Society of Hepatology (JSH) recommends screening every 3–4 months in patients with cirrhosis due to HBV or HCV and every six months in patients with other risk factors [44]. Despite these recommendations, HCC surveillance is not performed consistently. Systematic reviews report pooled surveillance utilization rates of 18.4% (95% CI: 17.8% – 19.0%) and 24.0% (95% CI 18.4% − 30.1%), highlighting substantial underuse [45, 46]. A retrospective cohort study of five US-based centers quantified the underutilization of biannual HCC surveillance at over 80%, with surveillance rates ranging from 5.3% to 33.3%[47].

### Racial and Ethnic Disparities in HCC Screening

Data on global racial/ethnic-based screening disparities are limited, and the data are largely United States (U.S.)-centric. Multiple studies found lower HCC surveillance among Black patients with cirrhosis, with 59% receiving no surveillance compared to 47% White and 49% Hispanic patients, and significantly reduced odds of surveillance across several cohorts (OR range 0.40–0.88) [47–50]. In a multicenter retrospective cohort study of patients with HCV cirrhosis, Black patients were less likely to receive routine HCC surveillance compared to White patients (OR: 0.60, 95% CI 0.45–0.81, *p*= 0.001)[26]. Conversely, a recent sample of 14,566 patients with cirrhosis across 4 different safety-net health systems found greater odds of HCC surveillance among racial/ethnic minorities when compared to non-Hispanic White patients (Black/African Americans: odds ratio [OR] 1.14, 95% confidence interval [CI] 1.01–1.28; Hispanics: OR 1.25, 95% CI 1.11–1.42; Asians: OR 1.70, 95% CI 1.32–2.17) [51]. This observation was attributed to affiliations with teaching hospitals, where there may be more outreach programs and greater staff adherence to surveillance guidelines.

###  Gender Disparities in HCC Screening

The data regarding gender-related HCC screening disparities appear discordant [45]. Many studies report higher surveillance rates among women compared to men [52–54]. In a sample of 1,873 patients with cirrhosis, 21.5% of women received regular surveillance for HCC, compared to 14.9% of men, p 0.006; however, this study was not balanced by gender [48]. Another study found that a higher proportion of women, 37.9%, were diagnosed with HCC through surveillance than men, 29.6%, who were more likely to be diagnosed due to symptoms (*p*= 0.003)[55]. Other studies found no statistically significant gender differences in HCC screening (*p* = 0.80 and *p*= 0.99)[56, 57].

### The Role of Socioeconomic Status on HCC Screening

Socioeconomic factors and financial burden may limit adherence to prescribed screening recommendations. In a survey investigating patient-reported financial burdens of HCC surveillance, 522 patients (51.2%) reported at least one barrier which included costs (28.9%), transportation difficulty (17.8%), and difficulty scheduling the ultrasound (24.1%)[58]. Underinsured patients consistently have lower rates of HCC surveillance (OR 1.43; 95% CI: 1.03–1.98) [49]. In a study of 1,873 patients with cirrhosis, patients living in areas with higher median income and a higher proportion of residents with greater than high school education were more likely to receive regular HCC surveillance vs. patients from regions with lower median income or less than a high school education (*P* < 0.001 and *P* = 0.003, respectively) [48]. Compared to individuals where < 5% of the population lived below the poverty line, individuals where > 25% of the population lived above the poverty line had significantly higher odds of HCC diagnosis in the emergency department (ED) (OR 1.42; 95% CI: 1.06–1.89, *p* = 0.019) and those who were uninsured had nearly 4-fold higher odds of being diagnosed in the ED rather than by screening when compared to those with private insurance (OR 3.85; 95% CI: 2.42–6.14, *p*< 0.001)[59]. Socioeconomic constraints also hinder HCC surveillance globally. In Sub-Saharan Africa where limited surveillance infrastructure, delayed referrals, and financial constraints contribute to late-stage presentation and poor outcomes [60, 61]. In Sweden, patients with cirrhosis and lower household income (first quartile across all households) were least likely to be diagnosed with HCC diagnosis through surveillance; 30% of low-income individuals were diagnosed through surveillance compared to 36% of medium-income (second and third quartile) individuals, and 38% of high-income (fourth quartile) individuals, *p*< 0.001[62].

## Disparities in HCC stage at diagnosis

Disparities in HCC screening translate to disparities in stage at presentation, treatment, and outcomes. The Barcelona Clinic Liver Cancer (BCLC) staging classification is the most widely used and guides prognosis and treatment [63, 64]. There are five stages of HCC: very early stage (BCLC 0), early stage (BCLC-A), intermediate stage (BCLC-B), advanced stage (BCLC-C), and end-stage (BCLC-D)[65]. See Table 1 below.

**Table 1:** Definition of Barcelona Clinic Liver Cancer Stage and Associated Prognosis

BCLC Stage	Definition	Prognosis (with appropriate treatment)
Very early stage (0)	Single ≤ 2 cm Preserved liver function PS 0	> 5 years
Early stage (A)	Single or ≤ 3 nodules, each ≤ 3 cm Preserved liver function PS 0	> 5 years
Intermediate stage (B)	Multinodular Preserved liver function PS 0	From > 5 years to > 2 years
Advanced stage (C)	Portal invasion or extrahepatic spread Preserved liver function PS 1–2	> 2 years
Terminal stage (D)	Any tumor burden	3 months

BCLC = Barcelona Clinic Liver Cancer, PS = Performance Status. Description and prognosis estimates derived from “BCLC Strategy for Prognosis Prediction and Treatment Recommendation: The 2022 Update.”[65]

There are limited global data on how stage at HCC diagnosis is distributed. The global HCC BRIDGE study, a longitudinal cohort study of 18,031 patients found that BCLC C stage at diagnosis was the most common stage in North America, Europe, China and South Korea with 42%, 51%, 55%, and 53% of patients presenting at that stage, respectively. Conversely, 70% of patients with HCC from Taiwan and 73% from Japan were diagnosed with BCLC 0/A stage disease [66]. As with disparities in HCC risk and screening, there are racial/ethnic, gender, and socioeconomic disparities in stage at HCC presentation.

When compared to patients who were diagnosed incidentally or due to symptoms, patients diagnosed with HCC via surveillance were significantly more likely to be diagnosed at earlier stage (Barcelona Clinic Liver Cancer (BCLC) stage 0 or A) (OR 2.56; 95% CI: 1.67–3.93), receive curative treatment (OR 2.03; 95% CI: 1.21–3.51), and experience improved survival (hazard ratio (HR) 0.69, 95% CI: 0.52–0.91) [47]. In another cohort, 63.1% of patients diagnosed with HCC by screening were diagnosed at BCLC stage 0 or A compared to 36.4% of those diagnosed incidentally, *p*< 0.001[67].

###  Racial and Ethnic Disparities in Stage at HCC Presentation

Multiple studies have highlighted significant racial disparities in cancer stage. Many patients with risk factors do not have access to regular screening and rely on the emergency department for health care. In a sample of 1,620 patients diagnosed with HCC in an emergency department (ED), patients were more likely to be Black (OR1 0.7; 95% CI: 1.2–2.3, *p* = 0.002) or Hispanic (OR 1.6; 95% CI: 1.1–2.6, *p* = 0.029) vs. White. Only 30.4% of Black patients with HCC were diagnosed with early-stage HCC (BCLC 0/A) compared to 54.4% of White patients, 46.8% of Hispanic patients, and 40.9% of Asian patients, *p*< 0.01[68]. Across two large health systems, Black (OR 0.74; 95% CI: 0.56–0.98) and Hispanic (OR 0.75; 95% CI, 0.55–1.00.55.00) patients were significantly less likely to be diagnosed with early-stage HCC compared to White patients [69–71]. A systematic review and meta-analysis comprising 563,097 HCC patients found that Black patients (OR 0.66; 95% CI: 0.54–0.78) had significantly lower odds of early-stage HCC compared to White patients, however there was no significant difference in stage among Asian, Hispanic or White patients [72]. 

A large SEER analysis found that Asian individuals were more likely to be diagnosed with larger tumors, with a median tumor size of 5.5 cm vs. 4.8 cm in White, 5.1 cm in Black and 4.8 cm in Native American individuals, *p*< 0.001[73]. However, a different retrospective analysis of the SEER database, comprising 50,723 patients with HCC between 2003 and 2011, reported that Black patients had significantly more advanced HCC at diagnosis. Despite the increased tumor size noted in Asian patients in the aforementioned SEER study, this study found that Asian patients were less likely to have advanced disease (OR 0.87; 95% CI: 0.80–0.94, *p* < 0.001) compared to White patients [71]. This discordance underscores the need for further analyses in diverse patient populations.

### Gender Differences in Stage at HCC Presentation

There are few studies that report on gender differences in HCC stage at diagnosis. A retrospective cohort study of 1,886 patients found that women were more likely to be diagnosed with BCLC A disease, 56.2%, compared to 41.8% of men, *p* < 0.001. Accordingly, men were more likely to be diagnosed with larger tumors and more tumors (3 or more), compared to women (27.6% vs. 22.5%, *p*= 0.04)[53]. This may be explained by previous observations that men are less likely to be diagnosed through routine surveillance. How the specific determinants driving gender disparities in HCC stage at diagnosis deserve further attention.

### Socioeconomic Differences in Stage at HCC Presentation

Socioeconomic factors such as marital status, insurance status, education level, and household income are associated with HCC stage [74]. Specifically, compared to patients with private insurance, patients with Medicaid had more advanced HCC at diagnosis (OR 1.29, 95% CI 1.11–1.49). Similarly, lower household income (<$40,000) was associated with greater odds of advanced stage HCC, OR 1.15; 95% CI: 1.01–1.32, p 0.04, compared to individuals in the highest income group (≥$70,000). Residing in rural areas (OR 1.10; 95% CI: 1.00–1.20.00.20; p 0.04) was associated with greater odds of advanced HCC at diagnosis vs. living in large metropolitan areas. Additionally, widowed patients had more advanced HCC diagnoses and ultimately worse prognoses; married patients were less likely to present with large tumors (> 5 cm) compared with widowed patients, *p*< 0.001[75].

## Disparities in the treatment and prognosis of HCC

**Fig. 3:**
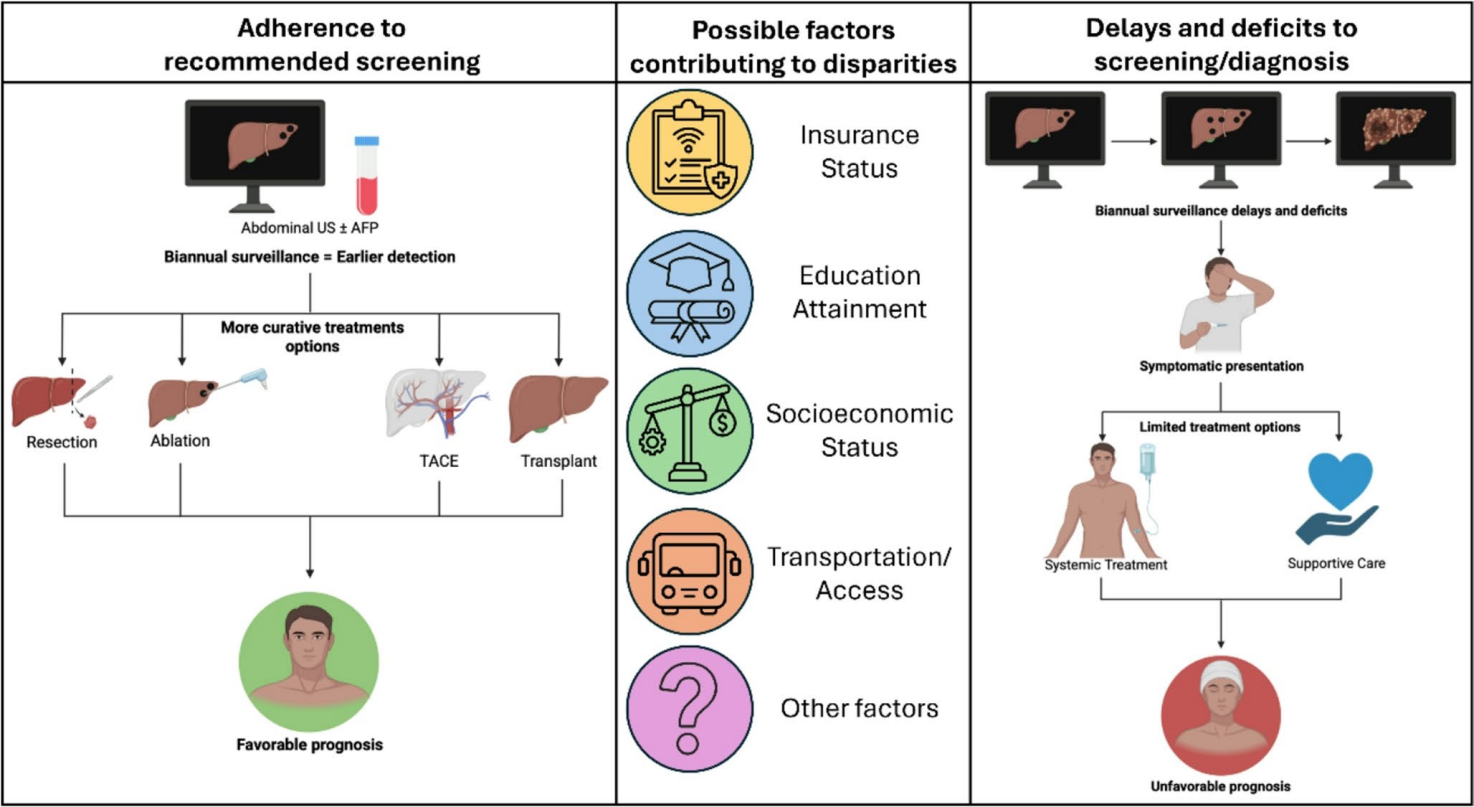
Schematic representation of the relationship between factors contributing to disparities with adherence to screening and delays in screening or diagnosis

Once a diagnosis of HCC is established, there are pervasive treatment disparities which impact prognosis. As with all cancers, prognosis is driven by tumor stage. However, the severity of liver dysfunction at the time of HCC diagnosis is an independent contributor to poor survival and directly influences the types of treatment offered and an individual’s ability to tolerate treatment [76]. Management options for HCC depend on cancer stage and include resection, ablation, transarterial chemoembolization (TACE), transarterial radioembolization (TARE), liver transplantation (LT), palliative care and symptomatic management. Although the BCLC model provides guidance regarding the intervention appropriate for each stage, many variables influence the treatments offered and received by a patient [65]. See Fig. 3 below. Patients diagnosed at an earlier stage are typically eligible for curative therapies with median survival exceeding 5 years, whereas patients diagnosed at later stages have a median survival of 12–18 months [72]. As a result of well-structured HCC surveillance programs, both Japan and Taiwan report significantly higher median survival [77].

In comparison, median survival after HCC diagnosis in sub-Saharan Africa is 2.5 months, which has been attributed to insufficient access to screening and limited treatment options [77]. 

In the early stage of HCC, overall survival depends on intervention offered and received. One study found that early-stage HCC detection (HR, 0.57; 95% CI, 0.54–0.60.54.60) and curative treatment receipt (HR, 0.42; 95% CI, 0.40‐0.44) are both associated with improved survival [78]. In a study performed in the U.S in patients with early stage HCC, 5-year survival ranged from 25% in patients who did not undergo any procedure to 77.3% patients who underwent transplant [79]. Once HCC has progressed to the advanced stages, systemic therapies, such as sorafenib, lenvatinib, atezolizumab-bevacizumab, and tremelimumab-durvalumab, have been approved in the United States by the FDA and may increase survival and improve morbidity [80]. Access, however, is uneven across and within countries including limited clinical trial availability, infrastructure/funding, and regulatory/reimbursement delays, all of which can delay access to therapies for months to years in these countries [80]. 

### Racial and Ethnic Disparities in HCC Treatment and Survival

In the U.S. and globally, Black patients experience significantly worse HCC survival [36, 68, 81]. This is driven partly by a higher likelihood of advanced stage presentation [82]. Compared with Non-Hispanic White patients, Black and Hispanic patients have lower odds of receiving HCC treatment (OR 0.77; 95% CI 0.77–0.81, *p* < 0.001 and OR 0.84; 95% CI 0.81–0.87; *p* < 0.001, respectively) and higher odds of delayed treatment (OR 1.12; 95% CI 1.05–1.19; *p* < 0.001 and OR 1.45; 95%CI 1.38–1.52; *p* < 0.001, respectively) [83]. Black patients with HCC at any stage are significantly less likely to receive any surgical intervention compared to White patients (19.8% vs. 22.9%, *p*< 0.001)[82]. Subsequently, Black patients also have a greater risk of death (HR 1.10; 95% CI 1.07–1.13; *p* < 0.001) compared to their Non-Hispanic White counterparts [83]. In a diverse sample of patients with HCC treated at a single center, median survival after HCC diagnosis was 425 days in Black patients with HCC compared to 904.5 days in White patients, *p*< 0.01[68].

Even when diagnosed at an early stage HCC, Black and Hispanic patients are less likely to receive curative treatment compared to White patients [70]. Black patients are less likely to undergo surgical resection for localized disease (adjusted RR 0.89, 95% CI: 0.84–0.94, *p* < 0.05) and lower odds of receiving curative treatment (OR, 0.76; 95% CI, 0.64–0.91.64.91) compared to White patients [78, 82]. Additionally, a statewide study in California found that Hispanic patients were less likely to receive treatment with curative intent compared to non-Hispanic White patients (OR 0.78; 95% CI 0.71–0.84; *p*< 0.001)[84]. In a large sample of 10,852 patients with HCC, only 4% of Black patients received transplant, compared to 7.5% of White patients, 9.3% of Hispanic patients, and 5.8% of Asian patients, *p*< 0.001[81]. Another study found that Black race was associated with lower transplantation rates with an adjusted odds ratio of 0.46 (95% CI: 0.42–0.51, *p* < 0.01) compared to White race [85]. Socioeconomic status likely plays a role in racial disparities in receipt of transplant. Both Black and Hispanic patients with low-SES have significantly lower rates of liver transplant (9.0% and 7.1%, respectively) compared to White patients with the same SES (12.2%), *p*< 0.05[79]. Fewer Black, 68.5%, and Hispanic, 66.7%, patients with low SES were treated at facilities that perform transplant, compared to 70.1% of White patients with low SES. Socioeconomic status likely limits access to other multidisciplinary care and clinical trials [79]. 

There are also disparities in receipt of systemic therapies. Compared to White patients, Hispanic (OR 0.63; 95% CI: 0.46–0.83; *p* < 0.01) and Black patients (OR 0.76; 95% CI: 0.65–0.88; *p* < 0.01) are less likely to receive immunotherapy [86, 87]. Similar to transplant, this may be related to treatment setting where Black and Hispanic patients are more likely to receive treatment. In academic settings, there were no significant differences in receipt of immunotherapy by race. However, in non-academic settings, Black patients (aOR: 0.48, 95% CI: 0.24–0.98) patients were significantly less likely to receive immunotherapy compared to White patients, p 0.023 [86]. 

While Hispanic individuals are less likely to receive resection or transplant when compared to non-Hispanic White individuals, multiple studies have demonstrated that there are not significant differences in median overall survival [78, 88]. Researchers have postulated that this is an example of the “Hispanic paradox,” an observation where Hispanic individuals have comparable or better survival rates than White individuals despite being more socioeconomically disadvantaged, having higher likelihood of advanced-stage HCC, and being less likely to receive treatment [72]. This survival advantage observed in Hispanic patients with HCC could also be partly attributed to differences in demographics, e.g. higher proportion of women, and/or the etiology of underlying liver disease [70]. In an analysis of Florida cancer registry data, Hispanic individuals the highest median survival time of 208 days, compared to 171 days in Whites, 158 days in Asians, and 136 days in Blacks, *p*< 0.001[81]. In this study, Hispanic patients lived closest to any liver transplant program, 10.6 miles, compared to 14.3 miles in Blacks, 15.2 miles in Asians, and 29.7 miles in Whites, *p*< 0.001[81]. Hispanics also lived closest to an academic cancer center, 16.3 miles, compared to 39.4 miles in Blacks, 54.3 miles in Asians, and 56.9 miles in Whites, *p*< 0.001[81]. Therefore, multiple factors likely interact to explain improved survival in the Hispanic population [81]. 

In aggregate, Asian and Pacific Islander or Native Hawaiian populations reported greater odds of receiving treatment (OR 1.40, 95% CI: 1.28–1.53, *p* < 0.001) for early-stage HCC and better overall survival (HR 0.75; 95% CI 0.71–0.79; *p*< 0.001)[84]. In another national study, API individuals were more likely to receive curative treatment, 34.4%, compared to 29.2% of White, 25.2% of Black, and 27.6% of AI/AN individuals, *p*< 0.001[73]. This same study also found that API individuals, had the lowest risk of death (HR 0.76; 95% CI 0.66–0.87, *p* < 0.001) compared to White individuals [73]. However, the Asian American, Native Hawaiian, and Pacific Islander (AANHPI) populations should not be treated as a homogenous cohort [89]. When disaggregated, South Asian patients were more likely to receive liver transplant compared to East Asian individuals, (HR 2.70; 95% CI 1.85–3.94; *p*< 0.01)[89]. Additionally, Southeast Asian (HR 1.43; 95% CI 1.22–1.68; *p* < 0.01) and Native Hawaiian and other Pacific Islander (HR 1.90, 95% CI 1.36–2.65; *p* < 0.01) individuals had higher mortality when compared with East Asian individuals [89]. Similar to studies demonstrating intragroup differences in HCC etiology, this study [89] highlights the need to disaggregate racial and ethnic groups which to inform culturally relevant cancer control and outreach efforts.

### Socioeconomic Disparities in HCC Treatment and Prognosis

Compared to patients with an annual household income of ≥ $70,000, patients with an income between $55,000 and $69,999 had significantly lower odds of receiving treatment (OR 0.90; 95% CI 0.88–0.93, *p*< 0.001)[83]. The odds of receiving treatment was even lower in patients with an income <$40,000 (OR 0.72; 95% CI 0.64–0.81; *p*< 0.001)[83]. Individuals with low SES were less likely to receive transplant or receive any procedures compared to individuals with high SES, *p*< 0.05[79]. Only 68.7% of low SES and 71.2% of middle SES individuals had access to specialized centers which offer transplant compared to 76.3% of high SES individuals, *p*< 0.05[79].

Medicaid insurance and uninsured status have consistently been associated with lower likelihood of receiving surgical treatment for curable early-stage HCC [90]. Medicaid insurance was associated with longer median time to transplant, 206 days, compared to 182 days in privately insured patients, *p*< 0.001[91]. Patients with Medicaid also have a higher risk of waitlist drop out due to death or declining clinical status which precludes transplant [91]. Of note, patients with Medicaid were more likely to be non-Hispanic Black or Hispanic in this study, which highlights the interplay between race, SES, and health insurance type that all may contribute to the disparities in HCC treatment and prognosis in these minority populations [91]. Disparities in SES impact treatment access on a global scale. In South Africa, more patients in the public sector received supportive care as their only treatment for HCC compared to those receiving care in the private sector (69.7% vs. 15.7%, *p*< 0.00001)[92]. The median survival was also lower in the patients treated in the public sector, 68 days, compared to private sector patients, 703 days, *p*< 0.001[92].

Along with disparities in access to facility type and treatment options, SES has been associated with increased mortality in HCC; compared to high-SES patients, 5-year overall survival was 7.0% lower for middle SES individuals and 11.2% lower for low SES individuals, *p*< 0.001[79]. When restricted to early-stage HCC, 5-year survival was 46.9% in patients with high SES, 39.9% in patients with middle SES, and 35.7% in patients with low SES, *p*< 0.001[79]. The only exception to this trend is noted among AI/AN individuals where SES did not impact survival [36]. 

### Geographic Disparities in HCC Treatment and Prognosis

Geographic factors contribute to barriers to care and worse prognosis for patients with HCC. In a California-based study, rural residence was associated with decreased odds of receiving curative therapy for early-stage HCC (OR 0.88, 95% CI 0.78–0.98, p 0.03) [84]. Receiving care at a non-National Cancer Institute(NCI)-designated cancer center also decreased the odds of receiving curative therapy (OR 0.48, 95% CI: 0.45–0.51, *p* < 0.001) and was associated with decreased survival (HR 1.33, 95% CI: 1.28–1.39, *p*< 0.001)[84]. Living 50 miles or further from the nearest transplant center was associated with decreased survival (HR 1.11; 95% CI 1.04–1.19, *p* < 0.003) when compared to those living within 20 miles [84]. Policies that address the gaps in healthcare access due to geography, such as increasing telehealth opportunities, improvement in transportation options, and increasing the number of transplant centers throughout the nation [93]. 

Individuals from rural areas experienced more delays in HCC treatment compared to those from metropolitan areas (OR 0.92, 95% CI: 0.84–1.00.84.00; p 0.047 [83]. Additionally, patients living in medium metropolitan areas (aHR, 1.09; 95% CI, 1.07–1.11; *P* < 0.001), small metropolitan areas (aHR, 1.08; 95% CI, 1.04–1.12, *p* < 0.001), and rural regions (aHR, 1.06; 95% CI, 1.02–1.09, p 0.004) all had significantly higher risk of mortality compared to patients from large metropolitan areas [83]. Geography, specifically living in rural areas, can further exacerbate racial and ethnic disparities in treatment and survival. When stratified by race, Black individuals from large metropolitan areas were 25% less likely (aOR 0.75; 95% CI, 0.71–0.80, *p* < 0.001) and Black individuals living in rural areas were 33% less likely to receive HCC treatment (aOR, 0.67; 95% CI, 0.57–0.79; *P* < 0.001), compared to White individuals living in large metropolitan areas [83]. 

## Conclusion

Disparities related to HCC are pervasive and can be attributed to multi-level factors at the patient, provider, and system levels. Along with country of origin, gender, and etiology of chronic liver disease, patient lifestyle, and access to healthcare influence risk of developing HCC and stage at diagnosis, which consequently influences treatment options and survival. To improve outcomes in the US and globally, it is critical that we focus on social determinants of health and eliminate barriers to equitable healthcare. We are in dire need of thoughtful evidence-based interventions, targeted especially at those populations which are impacted disproportionately, e.g. those living in rural communities, those without insurance coverage, or Black, Hispanic, and Asian communities with the greatest risk. Addressing health literacy, medical mistrust, access to healthcare and specialists, disseminating relevant information about disparities, and training healthcare providers to identify and mitigate potential implicit or explicit bias could help to eliminate disparities and positively shift the disease course for patients with HCC.

## Data Availability

No datasets were generated or analysed during the current study.

## Abbreviations

AAIR: age-adjusted incidence rate
aHR: adjusted hazard ratio
AI: American Indian
ALD: Alcohol-associated Liver Disease
AN: Alaska Native
aOR: adjusted odds ratio
APC: annual percent change
API: Asian/Pacific Islander
ASIR: age-standardized incidence rate
BCLC: Barcelona Clinic Liver Cancer
CI: confidence interval
EASL: European Association for the Study of the Liver
EASL-EORTC: European Association for the Study of the Liver-European Organization for Research and Treatment of Cancer
ED: Emergency Department
FDA: Food and Drug Administration
HBV: Hepatitis B virus
HCC: Hepatocellular Carcinoma
HCV: Hepatitis C virus
HDV: Hepatitis D virus
HIV: Human immunodeficiency virus
HR: hazard ratio
JSH: Japan Society of Hepatology
LT: liver transplantation
MASH: Metabolic Dysfunction-Associated Steatohepatitis
MASLD: Metabolic Dysfunction-Associated Steatotic Liver Disease
NIMHD: National Institute on Minority Health and Health Disparities
OR: odds ratio
PAF: population attributable fraction
RR: relative risk
SEER: Surveillance Epidemiology and End Results
SES: socioeconomic status
TACE: transarterial chemoembolization
TARE: transarterial radioembolization
US: United States
